# The effect of transverse ocular magnification adjustment on macular thickness profile in different refractive errors in community-based adults

**DOI:** 10.1371/journal.pone.0266909

**Published:** 2022-04-13

**Authors:** Hamed Niyazmand, Gareth Lingham, Paul G. Sanfilippo, Magdalena Blaszkowska, Maria Franchina, Seyhan Yazar, David Alonso-Caneiro, David A. Mackey, Samantha Sze-Yee Lee

**Affiliations:** 1 Division of Optometry, School of Allied Health, The University of Western Australia, Perth, WA, Australia; 2 Contact Lens and Visual Optic Laboratory, Centre for Vision and Eye Research, School, School of Optometry and Vision Science, Queensland University of Technology, Brisbane, QLD, Australia; 3 Lions Eye Institute, Centre for Ophthalmology and Visual Science, The University of Western Australia, Perth, WA, Australia; 4 Centre for Eye Research Ireland, School of Physics, Clinical and Optometric Sciences, Technological University Dublin, Ireland, Dublin, Ireland; 5 Centre for Eye Research Australia, University of Melbourne, Royal Victorian Eye and Ear Hospital, East Melbourne, VIC, Australia; 6 Garvan-Weizmann Centre for Cellular Genomics, Garvan Institute of Medical Research, Sydney, NSW, Australia; 7 School of Medicine, Menzies Research Institute Tasmania, University of Tasmania, Hobart, TAS, Australia; University of Houston, College of Optometry, UNITED STATES

## Abstract

**Purpose:**

Changes in retinal thickness are common in various ocular diseases. Transverse magnification due to differing ocular biometrics, in particular axial length, affects measurement of retinal thickness in different regions. This study evaluated the effect of axial length and refractive error on measured macular thickness in two community-based cohorts of healthy young adults.

**Methods:**

A total of 2160 eyes of 1247 community-based participants (18–30 years; 23.4% myopes, mean axial length = 23.6mm) were included in this analysis. Macular thickness measurements were obtained using a spectral-domain optical coherence tomography (which assumes an axial length of 24.385mm). Using a custom program, retinal thickness data were extracted at the 9 Early Treatment of Diabetic Retinopathy Study (ETDRS) regions with and without correction for transverse magnificent effects, with the corrected measurements adjusting according to the participant’s axial length. Linear mixed models were used to analyse the effect of correction and its interaction with axial length or refractive group on retinal thickness.

**Results:**

The raw measures (uncorrected for axial length) underestimated the true retinal thickness at the central macula, while overestimating at most non-central macular regions. There was an axial length by correction interaction effect in all but the nasal regions (all p<0.05). For each 1mm increase in axial length, the central macular thickness is overestimated by 2.7–2.9μm while thicknesses at other regions were underestimated by 0.2–4.1μm. Based on the raw thickness measurements, myopes have thinner retinas than non-myopes at most non-central macular. However, this difference was no longer significant when the corrected data was used.

**Conclusion:**

In a community-based sample, the raw measurements underestimate the retinal thickness at the central macula and overestimate the retinal thickness at non-central regions of the ETDRS grid. The effect of axial length and refractive error on retinal thickness is reduced after correcting for transverse magnification effects resulting from axial length differences.

## Introduction

The retinal thickness profile is a useful indicator in early detection and monitoring of various diseases [1, 2]. Using optical coherence tomography (OCT) imaging, central retinal thickening has been associated with exudative macular degeneration [3] and diabetic macular oedema [4], while thinning has been associated with loss of retinal nerve fibre layer (RNFL) [5, 6] and refractive error [7].

OCT imaging has been extensively used to assess the central and peripheral retinal thickness profile. Using OCT, changes in retinal thickness associated with axial length and/or refractive errors have been reported [8–11]; however, findings have not been always consistent. Studies have generally found that the central macular (fovea) thickness is positively associated with axial length, indicating myopic eyes exhibited a thicker central macula than non-myopic eyes [10, 12–19]. However, evaluation of the peri- and/or para-macular regions show conflicting findings; some studies have found thinner retinal thickness in inner and outer rings with axial elongation [8–12, 14, 15, 17–22] while others found no significant association [23–30]. In addition to considering the potential confounding factors such as the difference in the method of measuring retinal thickness, refractive error, and participants’ demographics of these studies, it is worth noting that some of these studies have not corrected for variations in the transverse magnification of the OCT images due to differences in ocular biometrics [8, 9, 12–22, 28, 30–32].

Variations in transverse magnification are largely due to differing axial lengths [33] and lead to changes in the transverse area of a pixel in the OCT image. Thus, while imaging eyes that are longer (more myopic) relative to the assumed axial length of the OCT system, a larger retinal area is captured. Since the retina is thickest at the parafoveal area and thins towards the fovea and the periphery, imaging of the retina over a larger area causes lower thickness values from more peripheral data points to be included in the analysis, while foveal thickness may be overestimated as the retina is thickest at the regions immediately surrounding the fovea (parafoveal regions). This leads to an underestimation of the peripheral macular thickness in longer eyes (e.g. in myopia), compared to those with shorter axial lengths (e.g. emmetropia and hyperopia). Using a Cirrus OCT, Odell et al. [34] found that the actual scan size of a 6 mm scan protocol may vary from 5.3 to 7.0 mm in a sample with axial length range 21.5 to 28.3 mm. This has practical implications, as altering the size of the OCT scanning area and other transverse boundaries results in under- or over-estimation of macular thickness. Thus, when assessing retinal thickness at a particular distance from the fovea, the placement of the measurement region should be adjusted to compensate for transverse magnification effects.

Unfortunately, many studies [8, 9, 12–22, 28, 30–32] do not account for transverse magnification effects when measuring retinal thickness with OCT, likely because there is usually no built-in function in OCT software to correct for participants’ ocular biometrics, including axial lengths. Some studies have corrected for transverse magnification using custom-written programs; however, such an approach is not readily available or feasible in the clinical practice [35–41]. The influence of transverse magnification correction on posterior tissue thickness measurements has been evaluated [35–38]; however, most have explored the effect of transverse magnification on the peripapillary retinal fibre layer thickness (RNFL) [35, 36, 38–41], or recruited mainly myopic participants and were thus are not representative of the general population [29]. There are limited population- or community-based studies exploring the effect of transverse magnification correction on retinal thickness or the association between axial length and retinal thickness, especially in a sample with a wide range of refractive errors.

Retinal thickness measurements may serve as an important biomarker in several ocular disease. However, accurate retinal thickness measurement is affected by transverse magnification effects, which are mainly caused by differing axial lengths. With myopia prevalence expected to increase in the next few decades [42]; it is becoming increasing critical to account for transverse magnification effects during OCT measurements. Given that axial length is a relatively easy measurement to obtain in a clinical setting, and has a major impact on transverse magnification, it may be justified to adjust for transverse magnification effects caused by differing axial lengths, where possible. To demonstrate how transverse magnification affects retinal thickness measurements, this study evaluated the effect of correction for transverse magnification based on differing axial lengths on two community-based samples with a wide range of refractive errors.

## Methods

### Study sample

Young healthy community-based adults were recruited from two cohort studies–Generation 2 (Gen2) of the Raine Study [43] and the Kidskin Young Adults Myopia Study (K-YAMS) [44]. Methodology for the eye examinations in both studies have been described previously [43, 44]. Briefly, for the Raine Study, 2,900 pregnant women were seen at the King Edward Memorial Hospital from May 1989 to November 1991, to whom 2,868 offspring were born, forming the original study cohort (termed “Gen2”). Since their birth, the Gen2 participants have been undergoing a series of health and medical examinations at various age. At the 20-year follow-up of the Raine Study between 2010 and 2012, the Gen2 participants had a comprehensive eye examination that included spectral-domain optical coherence tomography (SD-OCT) imaging.

The Kidskin Study was a non-randomised controlled trial in Western Australia that tested the value of an educational intervention on sun-protection behaviour in 5- to 6-year-old children in primary schools. The outcome measure was the number of melanocytic naevi on the backs of these children. Between 2015 and 2019, when they were 25–30 years of age, participants were invited to attend a comprehensive eye examination as part of the K-YAMS. The purpose of that eye examination was to explore whether these early educational interventions influence myopia development [45].

Both studies were conducted in accordance with the Declaration of Helsinki and were approved by the University of Western Australia Human Research Ethics Committee (Approval reference numbers RA/4/20/5722 and RA/4/1/6807) and all participants provided informed consent prior to data being collected.

### Eye examination

The eye examination included autorefraction and keratometry (Nidek ARK-510A Autorefractometer [Nidek Co Ltd, Tokyo, Japan]), ocular biometry (IOLMaster v 5 [Carl Zeiss Meditec AC, Jena, Germany]), and SD-OCT imaging Spectralis HRA+OCT [Heidelberg Engineering, Heidelberg, Germany]) [43, 44]. Autorefraction was taken at least 20 minutes after instillation of one drop of 1% tropicamide. The autorefractometer averages the measurements of three readings, and refraction was quantified using spherical equivalent, determined as spherical dioptre + ½ cylindrical dioptre. The IOLMaster calculates the mean of five A-scan measurements to obtain the axial length used for analyses. A-scan measurements that fall outside one standard deviation of the mean are removed from the computation.

A Spectralis spectral-domain OCT (SD-OCT) was used to image the macula, with an axial and transverse resolution of 3.9 and 5.6 μm, respectively. Macular thicknesses of both eyes were obtained using a 31-line raster scan of 30° horizontal and 25° vertical area centred on the fovea [46], obtained using high-resolution mode and with “Automated Real Time (ART)” of 9 taken per raster scan. Prior to imaging, the corneal radius was entered into the instrument to correct for ocular magnification effects due to corneal curvature, as per the manufacturer’s protocol. However, since the Heidelberg system does not provide the option to alter the axial length, all scans were taken with the default axial length of 24.385 mm, similar to the process in clinical practice. Scans with signal-to-noise ratio of less than 20 were considered to be of inadequate quality and were discarded from the analysis. Eyes with amblyopia, retinal disorders, and optic disc pathologies were excluded from the study.

**Fig 1:**
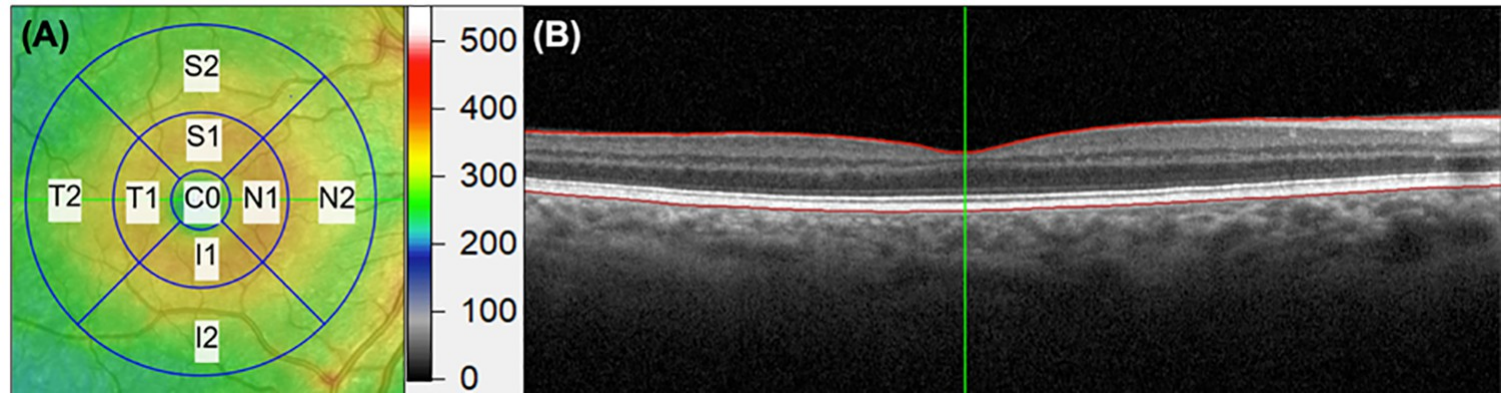
(A) The 9 Early Treatment of Diabetic Retinopathy Study (ETDRS) grid including central macula 0.5 mm radius around the fovea (C), the inner superior (S1), inner inferior (I1), inner temporal (T1), and inner nasal (N1) regions (between 0.5 and 1.5 mm radius around the fovea) and outer superior (S2), outer inferior (I2), outer temporal (T2), and outer nasal (N2) regions (between 1.5 and 3.0 mm radius around the fovea) macular rings. (B) A representative OCT B-scan with segmentation of the inner limiting membrane and retinal pigmented epithelium layer (red lines). Green line marks the foveal centre.

The raw SD-OCT scans were exported in E2E format using the built-in export function and analysed using a non-commercial custom program that was developed on MATLAB version R2017b (MathWorks, Inc. Natick, MA, USA) [46]. The transversal scaling (Scaling X) value for each eye, which differed between participants depending on the scan focus and corneal curvature adjustment, was extracted from the “image information” tab of the Heidelberg Eye Explorer. Using the Scaling X and axial length information, the program determines the lateral boundaries of the Early Treatment of Diabetic Retinopathy Study (ETDRS) grid (**Fig 1A**) by adjusting the transversal scale using the participant’s axial length and accounting for the Spectralis’ pre-set axial length of 24.385 mm [47], using the following equation:

Corrected transversal scale = Scan default transversal scale × (axial length/24.385).

The program detects the inner limiting membrane layer and retinal pigmented epithelium (**Fig 1B**), and the axial distance between these layers was determined as the full retinal thickness. The thickness values were extracted along each A-scan (axial thickness) [48] and averaged in each ETDRS region to provide the raw (uncorrected for axial length) and corrected retinal thickness.

### Statistical analysis

All statistical analyses were conducted using R software v3.6.3 (The R Foundation for Statistical Programming, Vienna, Austria; available at https://www.r-project.org/). The Kolmogorov–Smirnov test was used to confirm that none of the variables departed significantly from a normal distribution. A P value of <0.05 was considered statistically significant.

Linear mixed models (LMMs) were used to analyse the effect of -correction and its interaction with axial length on retinal thickness. The LMMs were generated with random intercepts and slopes for participants to account for the within-participant correlation between two eyes, and correction for gender. To analyse the effect of correction in different refractive groups (myopes; spherical equivalent of ≤ -0.50D vs. non-myopes) [49], separate LMMs were generated with refractive groups (myopes and non-myopes) included in the models instead of axial length. Given retinal thickness is known to vary between sexes [50] and possibly ethnicities [51, 52] these were adjusted for in the LMMs. Separate analyses were conducted for the Raine Study and K-YAMS cohorts to evaluate the agreeability between cohorts, as a sensitivity analysis. In all analyses, the restricted maximum likelihood methods were used, and pairwise comparisons with Bonferroni adjustments were conducted for any significant main effects and interactions.

## Results

### Demographic information

**Fig 2:**
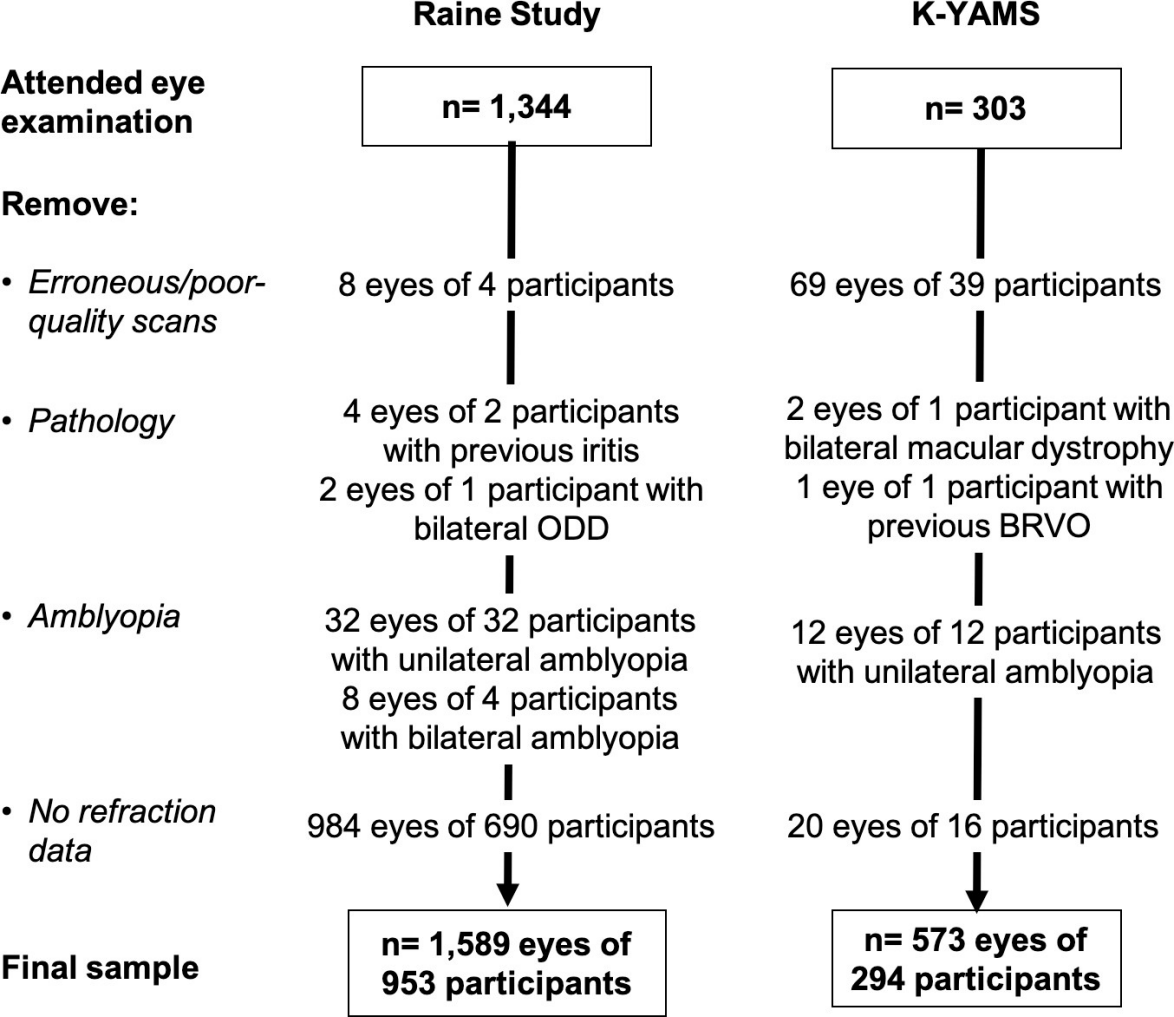
Sample size and reasons for exclusion from the data analysis in both the Raine Study and K-YAMS cohorts. BRVO = branch retinal vein occlusion, ODD = optic disc drusen.

**Fig 3:**
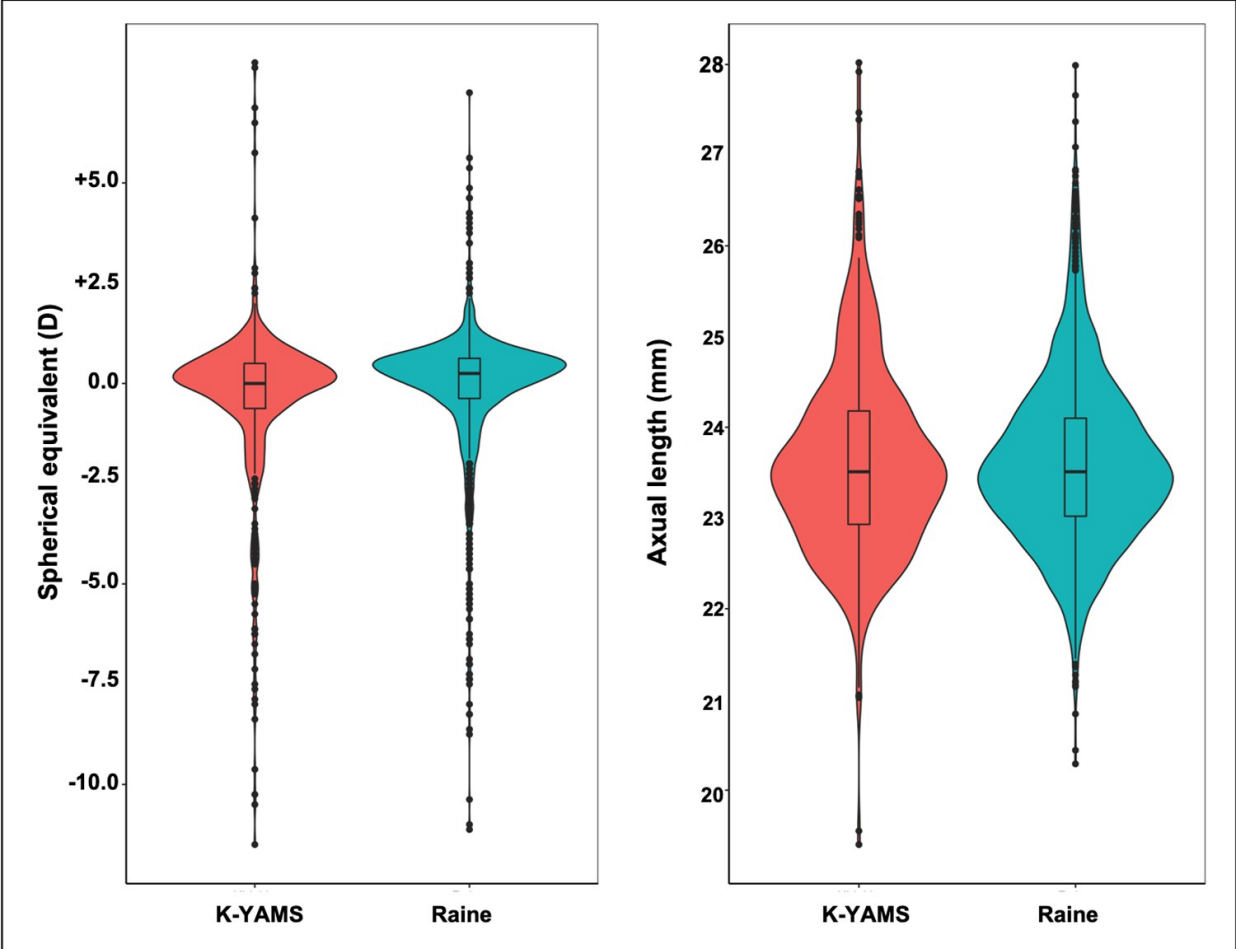
Distributions of refractive errors (D) and axial lengths (mm) in the Raine Study and K-YAMS cohorts.

A total of 1589 eyes of 953 participants from the Raine Study and 573 eyes of 294 participants from the K-YAMS were included in the analysis (see **Fig 2** for number of participants or eyes excluded along with reason for exclusion). Demographic and refractive information of the study cohorts are shown in **Table 1**. Distribution of refractive error and axial length are shown in **Fig 3**. The raw and -corrected retinal thickness values in myopes and non-myopes are shown in **S1 and S2 Figs.**

**Table 1:** Demographic information of the study participants included.

Factors	The Raine Study Gen2 (n = 953)	K-YAMS (n = 294)
Age (years)	20.1 ± 0.4 (18 to 22)	27.5 ± 1.1 (25 to 30)
Gender: female	467 (49%)	179 (61%)
Spherical equivalent (D)	-0.08 ± 1.55 (Range = -11.25 to +7.25)	-0.35 ± 1.95 (Range = -11.50 to +8.00)
Refractive group (number of eyes)		
• Myopes • Non-myopes	345 (22%) 1244 (78%)	160 (28%) 413 (72%)
Axial length (mm)	23.6 ± 0.9 (Range = 20.3 to 28.0)	23.6 ± 1.1 (Range = 19.4 to 28.0)
Ethnicity • Caucasian • East Asians • Other/mixed	820 (86%) 20 (2%) 113 (12%)	253 (86%) 3 (0.1%) 38 (13%)

K-YAMS = Kidskin Young Adult Myopia Study; continuous variables expressed as mean ± standard deviation (and range); categorical variables expressed as number and percentage

### Main effect of correction

**Fig 4:**
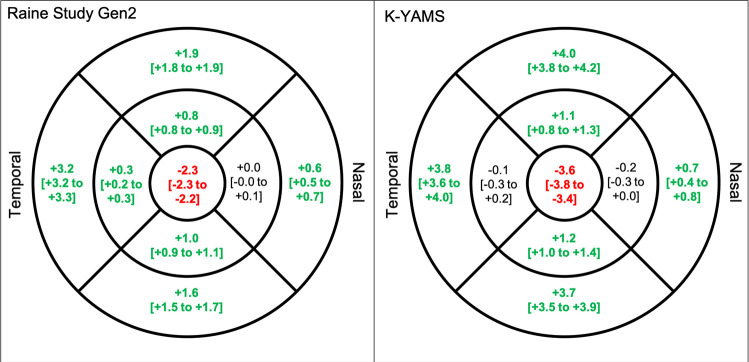
Estimated difference [and 95% confidence interval] in corrected and raw retinal thickness (μm) in (left) the Raine Study Gen 2 (mean axial length = 23.6 ± 0.9 mm) and (right) K-YAMS participants (mean axial length = 23.6 ± 1.1 mm). Significant difference (p< 0.05) shown in bold, with red and green values representing underestimation and overestimation by the raw measurements, relative to the transverse magnification-corrected retinal thickness, respectively. Adjusted for sex and ethnicity. Note that the mean axial lengths of both cohorts are shorter than that assumed by the Spectralis SD-OCT (24.385 mm).

There was a small but statistically significant difference in corrected and raw retinal thickness while adjusting for sex and ethnicity in most macular ETDRS regions (all p < 0.001). As shown in **Fig 4**, raw measures underestimated the retinal thickness at the central macula, while overestimating retinal thickness at most non-central macular regions in both the Raine and K-YAMS studies.

### Axial length by correction interaction effect

**Fig 5:**
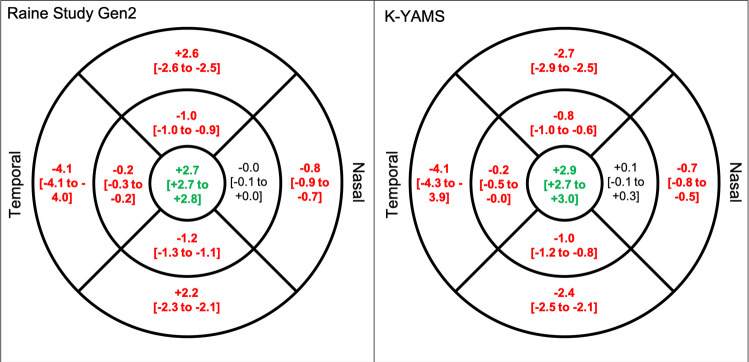
Under- and overestimation of retinal thickness (μm) per 1mm increase in axial length by the raw measurements, relative to the transverse magnification-corrected thickness [and 95% confidence interval] in (left) the Raine Study Gen2 (mean axial length = 23.6 ± 0.9 mm) and (right) K-YAMS participants (mean axial length = 23.6 ± 1.1 mm). Note that the mean axial length of both cohorts is shorter than that assumed by the Spectralis SD-OCT (24.385 mm). Significant interaction effects (p< 0.05) shown in bold and red (underestimation) or green (overestimation). Adjusted for sex and ethnicity.

There was a significant axial length-by-correction interaction effect in all but the nasal regions (all p < 0.05). **Fig 5** shows the axial length-by-correction interaction effect in each region while adjusting for sex and ethnicity in both the Raine Study and KYAMS cohorts. These findings indicate that for each 1mm increase in axial length, the central macular thickness is overestimated by 2.7 to 2.9μm when the measurements are not corrected for transverse magnification effects (**Fig 3**) but the thickness at the other regions is underestimated by 0.2 to 4.1μm, with the outer temporal region being most affected. As seen in **Fig 5**, the amount of under- or overestimation per 1mm change in axial length was very similar for both cohorts.

The simple effects of axial length on the raw and corrected retinal thickness were analysed separately to explore the effect of correction for transverse magnification on the previously reported association between axial length and retinal thickness.

Using the raw retinal measurements, each millimetre increase in axial length was associated with thinner retinal in the non-central region by -2.1 to -3.0 μm in the Raine Study Gen2 cohort and by -2.0 to -3.4 μm in the K-YAMS cohort (all p<0.001; **Table 2**). Using the corrected data, longer axial length remained significantly associated with thinner retinal thickness outside the central macula, albeit at generally smaller magnitudes than observed with the raw measurements (**Table 2**). These findings indicate that the effect of longer or shorter axial length on macular thickness may be smaller than previously reported, but still statistically significant. At the central macula, there was no association between retinal thickness and axial length found when using the raw measurements. However, a negative association between retinal thickness and axial length was observed in both cohorts with the corrected measurements. At the outer inferior, temporal and superior regions, longer axial length was associated with thinner retinal thickness before, but not with the corrected measurements.

**Table 2:** Estimated difference in retinal thickness (μm) per 1mm increase in axial length.

Macular region^a^	The Raine Study Gen2				K-YAMS			
	Raw		Corrected^b^		Raw		Corrected^b^	
	Estimate (95%CI)	p-value	Estimate (95%CI)	p-value	Estimate (95%CI)	p-value	Estimate (95%CI)	p-value
Central	0.2 [-1.1 to 1.4]	0.77	-2.5 [-3.7 to -1.2]	<0.001**	0.7 [-1.2 to 2.6]	0.47	-2.2 [-4.1 to -0.2]	0.029*
Nasal inner	-2.3 [-3.2 to -1.4]	<0.001**	-2.3 [-3.2 to -1.4]	<0.001**	-3.3 [-4.5 to -2.0]	<0.001**	-3.7 [-4.9 to -2.4]	<0.001**
Nasal outer	-2.8 [-3.8 to -1.9]	<0.001**	-1.9 [-2.8 to -0.9]	<0.001**	-3.3 [-4.5 to -2.0]	<0.001**	-2.8 [-4.1 to -1.6]	<0.001**
Superior inner	-2.5 [-3.3 to -1.6]	<0.001**	-1.5 [-2.4 to -0.6]	0.001**	-3.4 [-4.7 to -2.0]	<0.001**	-3.1 [-4.4 to -1.8]	<0.001**
Superior outer	-2.6 [-3.5 to -1.8]	<0.001**	0.0 [-0.9 to 0.8]	0.95	-2.6 [-3.9 to -1.3]	<0.001**	-0.1 [-1.4 to 1.2]	0.88
Temporal inner	-2.2 [-3.0 to -1.3]	<0.001**	-1.8 [-2.6 to -0.9]	<0.001**	-2.0 [-3.3 to -0.7]	0.002**	-2.3 [-3.5 to -1.1]	<0.001**
Temporal outer	-3.0 [-3.8 to -2.1]	<0.001**	1.2 [0.4 to 2.0]	0.003**	-2.6 [-3.8 to -1.4]	<0.001**	0.9 [-0.3 to 2.1]	0.13
Inferior inner	-2.4 [-3.4 to -1.4]	<0.001**	-1.2 [-2.2 to -0.2]	0.020*	-3.0 [-4.3 to -1.7]	<0.001**	-2.5 [-3.8 to -1.2]	<0.001**
Inferior outer	-2.1 [-3.2 to -1.1]	<0.001**	0.1 [-0.9 to 1.1]	0.87	-2.6 [-4.0 to -1.3]	<0.001**	-0.7 [-2.1 to 0.6]	0.28

CI = confidence interval; TM = transverse magnification.

^a^ Early Treatment of Diabetic Retinopathy regions

^b^corrected for transverse magnification effects due to differences in axial lengths. Statistically significant at

*p< 0.05 and

**p< 0.01

### Main effect of refractive error

**Table 3** shows the main effect of myopia, relative to no-myopia, using the raw and corrected macular thickness data. Using the raw data, in the Raine Study Gen2, myopes have thinner retinas compared to non-myopes at most non-central macular regions. However, this difference was no longer significant in most macular regions when the corrected data was used. In the K-YAMS cohort, based on the raw thickness, myopes have significantly thinner retinas at the outer temporal and outer superior than non-myopes (by -1.7 and -2.5 μm, respectively); however, this difference was not statistically significant based on the corrected thickness.

**Table 3:** The mean magnitude of the difference (in μm) and 95% confidence intervals (CI) in retinal thickness of myopes (spherical equivalent ≤ -0.50D), in comparison to non-myopes, based on the raw and corrected data from the Raine Study Gen2 and K-YAMS cohorts.

Macular region^a^	The Raine Study Gen2 cohort				K-YAMS cohort			
	Raw		Corrected^b^		Raw		Ccorrected^b^	
	Estimate [95%CI]	P-value	Estimate [95%CI]	P-value	Estimate [95%CI]	P-value	Estimate [95%CI]	P-value
Central	1.0 [-1.1 to 3.2]	0.34	0.5 [-1.6 to 2.6]	0.64	-0.63 [-3.3 to 2.0]	0.64	-2.3 [-5.0 to 0.4]	0.09
Nasal inner	-1.4 [-2.7 to -0.1]	0.041*	-1.6 [-3.0 to -0.2]	0.024	-0.66 [-1.1 to 2.5]	0.47	-1.8 [-3.5 to -0.1]	0.035
Nasal outer	-2.5 [-4.0 to -1.1]	0.001**	-2.1 [-3.6 to -0.5]	0.010*	-0.10 [-1.8 to 1.6]	0.91	-0.3 [-1.9 to 1.3]	0.72
Superior inner	-1.9 [-3.3 to -0.5]	0.007**	-1.5 [-2.8 to -0.1]	0.040*	-1.72 [-3.6 to 0.2]	0.09	-1.6 [-3.3 to 0.1]	0.06
Superior outer	-2.7 [-4.2 to 1.3]	<0.001**	-1.0 [-2.5 to 0.4]	0.17	-2.48 [-1.5 to -0.5]	0.017*	-1.4 [-3.4 to -0.5]	0.15
Temporal inner	-0.7 [-2.0 to 0.6]	0.29	-0.95 [-2.4 to 0.5]	0.20	1.09 [-0.7 to 2.9]	0.23	-1.5 [-3.0 to 0.1]	0.07
Temporal outer	-3.1 [-4.6 to -1.5]	<0.001**	-0.2 [-1.7 to 1.4]	0.83	-2.24 [-4.0 to -0.4]	0.016*	-0.6 [-2.3 to 1.1]	0.47
Inferior inner	-3.2 [-5.3 to -1.1]	0.003**	-2.1 [-4.0 to -0.1]	0.039*	-1.75 [-0.2 to 3.6]	0.06	-1.6 [-3.4 to 0.2]	0.08
Inferior outer	-3.1 [-5.1 to -1.0]	0.004**	-1.0 [-3.0 to 1.0]	0.34	-1.69 [-3.6 to 0.3]	0.09	-0.8 [-2.7 to 1.2]	0.45

CI = confidence interval; K-YAMS = Kidskin Young Adult Myopia Study; TM

^a^ Early Treatment of Diabetic Retinopathy regions

^b^corrected for transverse magnification effects due to differences in axial lengths. Statistically significant at

*p< 0.05 and

**p< 0.01

## Discussion

This study evaluated the effect of transverse magnification due to differing axial lengths on the macular thickness in the ETDRS grid in two community-based cohorts with a wide range of refractive errors. This study found that, on average, the raw OCT measurements (which assume a more myopic sample than the community-based cohorts) underestimates the retinal thickness at the central macula and overestimate the retinal thickness at non-central regions. Moreover, the main effect of axial length and refractive error disappears or reduces after correction in most of the non-central macular regions. On the other hand, in the central macula, there was an inverse association between corrected thickness measurements and axial length, which was not observed when the raw measurements were used. It is worth noting that the Spectralis SD-OCT used in this study assumes a default axial length of 24.385 mm, which is longer than the mean axial length of 23.60 mm of the participants in this study. This means that the raw OCT measures were averaged over a smaller area (< 6mm diameter) in the current cohorts, result in an underestimation of the retinal thickness at the central macular and overestimation at the more peripheral regions. However, the effect of transverse magnification on an individual’s retinal thickness measurements would depend on the relation between participant’s axial lengths (and other ocular biometrics measures) and that assumed by the OCT system. For example, an opposite effect would be found in eyes longer than 24.385 mm, with the raw measurements providing an overestimation of the retinal thickness at the central macula but an underestimation at the non-central macular.

A comparison on the estimated effect on the association between axial length and retinal thickness between the raw and corrected data indicated that the main effect of axial length on retinal thickness was weaker with corrected data, relative to the raw measurements. Nonetheless, the association between axial length and retinal thickness remained significant in most macular regions after correcting for transverse magnification. These results demonstrated that although some, but not all, of the differences retinal thickness found between eyes with and without myopia could be attributed to the miscalculation of the retinal thickness, the axial length still has a significant effect on retinal thickness.

Results from this study showed differences in retinal thickness between myopic and non-myopic eyes is reduced (depending on the region) after correction for transverse magnification due to differing axial lengths. Consistent with our findings, Liu et al. [29] initially found a significant difference in central retinal thickness between myopes and hyperopes but further analyses showed no significant difference between refractive groups after adjusting for ocular magnification.

Transverse magnification effects have also been shown to affect peripapillary RNFL thickness measurements [36, 38–41]. A disc-centred scan would result in a smaller scan area in smaller eyes, thus over-estimating RNFL thickness because of the higher density of nerve fibres closer to the optic disc. In eyes with longer axial lengths, the scan area would be larger than the intended protocol, leading to an under-estimation of RNFL thickness. Hirasawa et al. [35] evaluated the peripapillary RNFL thickness in eyes with axial lengths of 21.2 to 28.3 mm and found that for every millimetre increase in axial length, the global RNFL thickness was under-estimated by 1.2μm. Other studies evaluating the effect of ocular magnification correction on RNFL thickness also found an inverse correlation between axial length and RNFL thickness which is not significant after correction of ocular magnification [36, 40, 41]. It is worth noting that the RNFL thickness, as evaluated by Hirasawa et al., is thinner that the total retinal thickness and thus a smaller magnitude of under- or overestimation in tissue thickness with each millimetre change in the axial length is expected in their study compare to our findings. Given the importance of retinal fibre layer thickness follow-up in glaucoma, further exploration of the effect of transverse magnification adjustment on peripapillary RNFL thickness may be especially relevant in younger populations. For example, when monitoring young adults or children with juvenile or secondary glaucoma, refractive error is likely to change as the eye elongates. Thus, without transverse magnification correction, the RNFL thickness is likely to appear to be thinning faster than it actually is as the eye elongates.

Commercially available OCT instruments are usually calibrated to a predetermined default axial length of around 24.5 mm (e.g. Cirrus, Spectralis, and Copernicus OCT instruments axial length default is 24.46, 24.38, and 24.00 mm, respectively), which generally provide reliable thickness measurement in emmetropic or low myopic eyes; however, elongated or shortened eyes required some adjustment for transverse magnification to cover the same scan area. Results from this study confirm that the axial length plays a key role in measurements and calculations of the retinal thickness profile using OCT, and adjustment for transverse magnification is something that OCT manufacturers could consider incorporating in their software. Most OCT devices’ software already allows the input of corneal curvature to correct for some of the magnification effects. Including axial length correction in the OCT instrument’s software prior to imaging will improve the accuracy of their measurements. The correction for transverse magnification due to differing axial lengths will become more crucial as almost half of the global population becomes myopic by year 2050 [42] and the accuracy of the retinal and choroidal thickness in high myopes may provide further insight into the mechanism underlying myopia development.

This study included a wide range of refractive errors, which allows evaluation of different refractive errors/axial length values on macular thickness in relation to transverse magnification. Another strength of the current study is that the Raine Study participants have been shown to be generally representative of the Western Australia young adult population in terms of socio-economic/demography [53]. The inclusion of two cohorts analysed separately served as a sensitivity analysis and allowed us to validate the findings from each study. A main limitation of this study is that it only included young adults. More studies are perhaps needed on other age groups and in people with various ocular diseases to provide further insight into the potential effects of the transverse magnification on posterior tissue thickness calculation using OCT. Another limitation of the study is that the custom program does not account for lens thickness or anterior chamber depth in the corrected measurements. However, axial length is the major driver behind transverse magnification effects [33].

In conclusion, results from this study show raw OCT measures of retinal thickness in the ETDRS grid underestimate the retinal thickness at the central macula and overestimate the retinal thickness at non-central regions. Moreover, the main effect of axial length and refractive error disappear or reduce after correction for transverse magnification due to differences in axial length in most of the non-central macular regions. Although the effect size found in this study seems rather small, as the world population becomes increasingly myopic, the results of this study will be more meaningful in the future. Hence, commercial OCTs should consider incorporating axial length in calculation of thickness measurements.

## Supporting information

S1 Fig Uncorrected full retinal thickness (μm) in the all (top row), myopic (middle row), and non-myopic (bottom row) participants in the Raine Study (left) and K-YAMS (right) cohorts, expressed in terms of median (in bold) [and interquartile range]. (DOCX)

S2 Fig Transverse magnification-corrected full retinal thickness (μm) in the all (top row), myopic (middle row), and non-myopic (bottom row) participants in the Raine Study (left) and K-YAMS (right) cohorts, expressed in terms of median (in bold) [and interquartile range]. (PDF)

## References

[1] Phadikar P, Saxena S, Ruia S, Lai TY, Meyer CH, Eliott D. The potential of spectral domain optical coherence tomography imaging based retinal biomarkers. *Int J Retina Vitreous*. 2017;3(1):1–10. doi: 10.1186/s40942-016-0054-7 28078103 PMC5220620

[2] Huang D, Swanson EA, Lin CP, et al. Optical coherence tomography. *Science*. 1991;254(5035):1178–1181. doi: 10.1126/science.1957169 1957169 PMC4638169

[3] Coscas G, Coscas F, Vismara S, Souied E, Soubrane G. Spectral Domain OCT in age-related macular degeneration: preliminary results with Spectralis HRA-OCT. *Journal francais d’ophtalmologie*. 2008;31(4):353–361. doi: 10.1016/s0181-5512(08)71429-3 18563034

[4] Ünsal E, Eltutar K, Zirtiloğlu S, Dinçer N, Erkul SÖ, Güngel H. Choroidal thickness in patients with diabetic retinopathy. *Clin Ophthalmol*. 2014;8:637. doi: 10.2147/OPTH.S59395 24707168 PMC3971934

[5] Horn FK, Mardin CY, Laemmer R, et al. Correlation between local glaucomatous visual field defects and loss of nerve fiber layer thickness measured with polarimetry and spectral domain OCT. *Invest Ophthalmol Vis Sci*. 2009;50(5):1971–1977. doi: 10.1167/iovs.08-2405 19151389

[6] El Beltagi TA, Bowd C, Boden C, et al. Retinal nerve fiber layer thickness measured with optical coherence tomography is related to visual function in glaucomatous eyes. *Ophthalmology*. 2003;110(11):2185–2191. doi: 10.1016/S0161-6420(03)00860-1 14597528

[7] Ohno-Matsui K, Lai TY, Lai C-C, Cheung CMG. Updates of pathologic myopia. *Prog Retin Eye Res*. 2016;52:156–187. doi: 10.1016/j.preteyeres.2015.12.001 26769165

[8] Luo H-D, Gazzard G, Fong A, et al. Myopia, axial length, and OCT characteristics of the macula in Singaporean children. *Invest Ophthalmol Vis Sci*. 2006;47(7):2773–2781. doi: 10.1167/iovs.05-1380 16799013

[9] Huynh SC, Wang XY, Rochtchina E, Mitchell P. Distribution of macular thickness by optical coherence tomography: findings from a population-based study of 6-year-old children. *Invest Ophthalmol Vis Sci*. 2006;47(6):2351–2357. doi: 10.1167/iovs.05-1396 16723444

[10] Lim MC, Hoh S-T, Foster PJ, et al. Use of optical coherence tomography to assess variations in macular retinal thickness in myopia. *Invest Ophthalmol Vis Sci*. 2005;46(3):974–978. doi: 10.1167/iovs.04-0828 15728555

[11] Takeyama A, Kita Y, Kita R, Tomita G. Influence of axial length on ganglion cell complex (GCC) thickness and on GCC thickness to retinal thickness ratios in young adults. *Jpn J Ophthalmol*. 2014;58(1):86–93. doi: 10.1007/s10384-013-0292-2 24242185

[12] Cheng SC, Lam CS, Yap MK. Retinal thickness in myopic and non‐myopic eyes. *Ophthal Physiol Opt*. 2010;30(6):776–784. doi: 10.1111/j.1475-1313.2010.00788.x 21205263

[13] Duan XR, Liang YB, Friedman DS, et al. Normal macular thickness measurements using optical coherence tomography in healthy eyes of adult Chinese persons: the Handan Eye Study. *Ophthalmology*. 2010;117(8):1585–1594. doi: 10.1016/j.ophtha.2009.12.036 20472290

[14] Wu P, Chen Y, Chen C, et al. Assessment of macular retinal thickness and volume in normal eyes and highly myopic eyes with third-generation optical coherence tomography. *Eye*. 2008;22(4):551–555. doi: 10.1038/sj.eye.6702789 17464309

[15] Harb E, Hyman L, Fazzari M, Gwiazda J, Marsh-Tootle W. Factors associated with macular thickness in the COMET myopic cohort. *Optom Vis Sci*. 2012;89(5):620. doi: 10.1097/OPX.0b013e318251293a 22525127 PMC3348261

[16] Wong A, Chan C, Hui S. Relationship of gender, body mass index, and axial length with central retinal thickness using optical coherence tomography. *Eye*. 2005;19(3):292–297. doi: 10.1038/sj.eye.6701466 15258609

[17] Lam DSC, Leung KS, Mohamed S, et al. Regional variations in the relationship between macular thickness measurements and myopia. *Invest Ophthalmol Vis Sci*. 2007;48(1):376–382. doi: 10.1167/iovs.06-0426 17197557

[18] Hwang YH, Kim YY. Macular thickness and volume of myopic eyes measured using spectral‐domain optical coherence tomography. *Clin Exp Optom*. 2012;95(5):492–498. doi: 10.1111/j.1444-0938.2012.00749.x 22759271

[19] Chen S, Wang B, Dong N, Ren X, Zhang T, Xiao L. Macular measurements using spectral-domain optical coherence tomography in Chinese myopic children. *Invest Ophthalmol Vis Sci*. 2014;55(11):7410–7416. doi: 10.1167/iovs.14-13894 25316719

[20] Song WK, Lee SC, Lee ES, Kim CY, Kim SS. Macular thickness variations with sex, age, and axial length in healthy subjects: a spectral domain–optical coherence tomography study. *Invest Ophthalmol Vis Sci*. 2010;51(8):3913–3918. doi: 10.1167/iovs.09-4189 20357206

[21] Jonas JB, Xu L, Wei WB, et al. Retinal thickness and axial length. *Invest Ophthalmol Vis Sci*. 2016;57(4):1791–1797. doi: 10.1167/iovs.15-18529 27074383

[22] Jin P, Zou H, Zhu J, et al. Choroidal and retinal thickness in children with different refractive status measured by swept-source optical coherence tomography. *Am J Ophthalmol*. 2016;168:164–176. doi: 10.1016/j.ajo.2016.05.008 27189931

[23] Kelty PJ, Payne JF, Trivedi RH, Kelty J, Bowie EM, Burger BM. Macular thickness assessment in healthy eyes based on ethnicity using Stratus OCT optical coherence tomography. *Invest Ophthalmol Vis Sci*. 2008;49(6):2668–2672. doi: 10.1167/iovs.07-1000 18515595

[24] Wakitani Y, Sasoh M, Sugimoto M, Ito Y, Ido M, Uji Y. Macular thickness measurements in healthy subjects with different axial lengths using optical coherence tomography. *Retina*. 2003;23(2):177–182. doi: 10.1097/00006982-200304000-00007 12707596

[25] Ooto S, Hangai M, Tomidokoro A, et al. Effects of age, sex, and axial length on the three-dimensional profile of normal macular layer structures. *Invest Ophthalmol Vis Sci*. 2011;52(12):8769–8779. doi: 10.1167/iovs.11-8388 21989721

[26] Ooto S, Hangai M, Sakamoto A, et al. Three-dimensional profile of macular retinal thickness in normal Japanese eyes. *Invest Ophthalmol Vis Sci*. 2010;51(1):465–473. doi: 10.1167/iovs.09-4047 19696169

[27] Chan C-M, Yu J-H, Chen L-J, et al. Posterior pole retinal thickness measurements by the retinal thickness analyzer in healthy Chinese subjects. *Retina*. 2006;26(2):176–181. doi: 10.1097/00006982-200602000-00009 16467674

[28] Zou H, Zhang X, Xu X, Yu S. Quantitative in vivo retinal thickness measurement in chinese healthy subjects with retinal thickness analyzer. *Invest Ophthalmol Vis Sci*. 2006;47(1):341–347. doi: 10.1167/iovs.05-0480 16384983

[29] Liu X, Shen M, Yuan Y, et al. Macular thickness profiles of intraretinal layers in myopia evaluated by ultrahigh-resolution optical coherence tomography. *Am J Ophthalmol*. 2015;160(1):53–61. e52. doi: 10.1016/j.ajo.2015.03.012 25800454

[30] Barrio‐Barrio J, Noval S, Galdós M, et al. Multicenter Spanish study of spectral‐domain optical coherence tomography in normal children. *Acta Ophthalmologica*. 2013;91(1):e56–e63. doi: 10.1111/j.1755-3768.2012.02562.x 23347665

[31] Yanni SE, Wang J, Cheng CS, et al. Normative reference ranges for the retinal nerve fiber layer, macula, and retinal layer thicknesses in children. *Am J Ophthalmol*. 2013;155(2):354–360. e351. doi: 10.1016/j.ajo.2012.08.010 23127751 PMC3545013

[32] Nieves-Moreno M, Martínez-de-la-Casa JM, Morales-Fernández L, Sánchez-Jean R, Sáenz-Francés F, García-Feijoó J. Impacts of age and sex on retinal layer thicknesses measured by spectral domain optical coherence tomography with Spectralis. *PloS One*. 2018;13(3):e0194169. doi: 10.1371/journal.pone.0194169 29522565 PMC5844598

[33] Garway-Heath DF, Rudnicka AR, Lowe T, Foster PJ, Fitzke FW, Hitchings RA. Measurement of optic disc size: equivalence of methods to correct for ocular magnification. *Br J Ophthalmol*. 1998;82(6):643–649. doi: 10.1136/bjo.82.6.643 9797665 PMC1722616

[34] Odell D, Dubis AM, Lever JF, Stepien KE, Carroll J. Assessing errors inherent in OCT-derived macular thickness maps. *Ophthalmology*. 2011;2011. doi: 10.1155/2011/692574 21869920 PMC3157761

[35] Hirasawa K, Shoji N, Yoshii Y, Haraguchi S. Determination of axial length requiring adjustment of measured circumpapillary retinal nerve fiber layer thickness for ocular magnification. *PloS One*. 2014;9(9):e107553. doi: 10.1371/journal.pone.0107553 25215521 PMC4162641

[36] Aykut V, Öner V, Taş M, İşcan Y, Ağaçhan A. Influence of axial length on peripapillary retinal nerve fiber layer thickness in children: a study by RTVue spectral-domain optical coherence tomography. *Curr Eye Res*. 2013;38(12):1241–1247. doi: 10.3109/02713683.2013.820328 23972028

[37] Sanchez‐Cano A, Baraibar B, Pablo LE, Honrubia FM. Magnification characteristics of the optical coherence tomograph STRATUS OCT 3000. *Ophthal Physiol Opt*. 2008;28(1):21–28. doi: 10.1111/j.1475-1313.2007.00527.x 18201332

[38] Savini G, Barboni P, Parisi V, Carbonelli M. The influence of axial length on retinal nerve fibre layer thickness and optic-disc size measurements by spectral-domain OCT. *Br J Ophthalmol*. 2012;96(1):57–61. doi: 10.1136/bjo.2010.196782 21349942

[39] Öner V, Aykut V, Taş M, Alakuş MF, İşcan Y. Effect of refractive status on peripapillary retinal nerve fibre layer thickness: a study by RTVue spectral domain optical coherence tomography. *Br J Ophthalmol*. 2013;97(1):75–79. doi: 10.1136/bjophthalmol-2012-301865 23143906

[40] Öner V, Taş M, Türkcü FM, Alakuş MF, İşcan Y, Yazıcı AT. Evaluation of peripapillary retinal nerve fiber layer thickness of myopic and hyperopic patients: a controlled study by Stratus optical coherence tomography. *Curr Eye Res*. 2013;38(1):102–107. doi: 10.3109/02713683.2012.715714 22913797

[41] Bayraktar S, Bayraktar Z, Yilmaz ÖF. Influence of scan radius correction for ocular magnification and relationship between scan radius with retinal nerve fiber layer thickness measured by optical coherence tomography. *J Glaucoma*. 2001;10(3):163–169. doi: 10.1097/00061198-200106000-00004 11442177

[42] Holden BA, Fricke TR, Wilson DA, et al. Global prevalence of myopia and high myopia and temporal trends from 2000 through 2050. *Ophthalmology*. 2016;123(5):1036–1042. doi: 10.1016/j.ophtha.2016.01.006 26875007

[43] Yazar S, Forward H, McKnight CM, et al. Raine eye health study: design, methodology and baseline prevalence of ophthalmic disease in a birth-cohort study of young adults. *Ophthalmic Genet*. 2013;34(4):199–208. doi: 10.3109/13816810.2012.755632 23301674

[44] Lingham G, Milne E, Cross D, et al. Investigating the long-term impact of a childhood sun-exposure intervention, with a focus on eye health: protocol for the Kidskin-Young Adult Myopia Study. *BMJ open*. 2018;8(1):e020868. doi: 10.1136/bmjopen-2017-020868 29391375 PMC5829843

[45] Lingham G, Yazar S, Lucas RM, et al. Time spent outdoors in childhood is associated with reduced risk of myopia as an adult. *Sci Rep*. 2021;11(1):6337. doi: 10.1038/s41598-021-85825-y 33737652 PMC7973740

[46] Lee SS-Y, Lingham G, Alonso-Caneiro D, et al. Macular Thickness Profile and Its Association With Best-Corrected Visual Acuity in Healthy Young Adults. *Translational vision science & technology*. 2021;10(3):8–8. doi: 10.1167/tvst.10.3.8 34003942 PMC7961121

[47] Ctori I, Gruppetta S, Huntjens B. The effects of ocular magnification on Spectralis spectral domain optical coherence tomography scan length. *Graefe’s Arch Clin Exp Ophthalmol*. 2015;253(5):733–738. doi: 10.1007/s00417-014-2915-9 25572356

[48] Kugelman J, Alonso-Caneiro D, Read SA, et al. Automatic choroidal segmentation in OCT images using supervised deep learning methods. *Scientific Reports*. 2019;9(1):13298. doi: 10.1038/s41598-019-49816-4 31527630 PMC6746702

[49] Flitcroft DI, He M, Jonas JB, et al. IMI—Defining and Classifying Myopia: A Proposed Set of Standards for Clinical and Epidemiologic Studies. *Invest Ophthalmol Vis Sci*. 2019;60(3):M20–M30. doi: 10.1167/iovs.18-25957 30817826 PMC6735818

[50] Lee SS-Y, Lingham G, Alonso-Caneiro D, et al. Macular thickness profile and its association with best-corrected visual acuity in healthy young adults. *Transla Vis Sci Technol*. 2021;10(3):8–8. doi: 10.1167/tvst.10.3.8 34003942 PMC7961121

[51] Kashani AH, Zimmer-Galler IE, Shah SM, et al. Retinal thickness analysis by race, gender, and age using Stratus OCT. *Am J Ophthalmol*. 2010;149(3):496–502. e491. doi: 10.1016/j.ajo.2009.09.025 20042179 PMC2826608

[52] Pilat AV, Proudlock FA, Mohammad S, Gottlob I. Normal macular structure measured with optical coherence tomography across ethnicity. *Br J Ophthalmol*. 2014;98(7):941–945. doi: 10.1136/bjophthalmol-2013-303119 24518076

[53] Straker L, Mountain J, Jacques A, et al. Cohort profile: the Western Australian pregnancy cohort (raine) study–generation 2. *Int J Epidemiol*. 2017;46(5):1384–1385. doi: 10.1093/ije/dyw308 28064197 PMC5837608

