# Supplementary material for: The effect of transverse ocular magnification adjustment on macular thickness profile in different refractive errors in community-based adults
Source: PLoS One. 2022 Apr 13;17(4):e0266909. doi: 10.1371/journal.pone.0266909 (PMC9007368; doi:10.1371/journal.pone.0266909)

Supplementary Figure 2. **Transverse magnification-corrected full retinal thickness** (μm) in the all (top row), myopic (middle row), and non-myopic (bottom row) participants in the Raine Study (left) and K-YAMS (right) cohorts, expressed in terms of **median (in bold)** [and interquartile range].

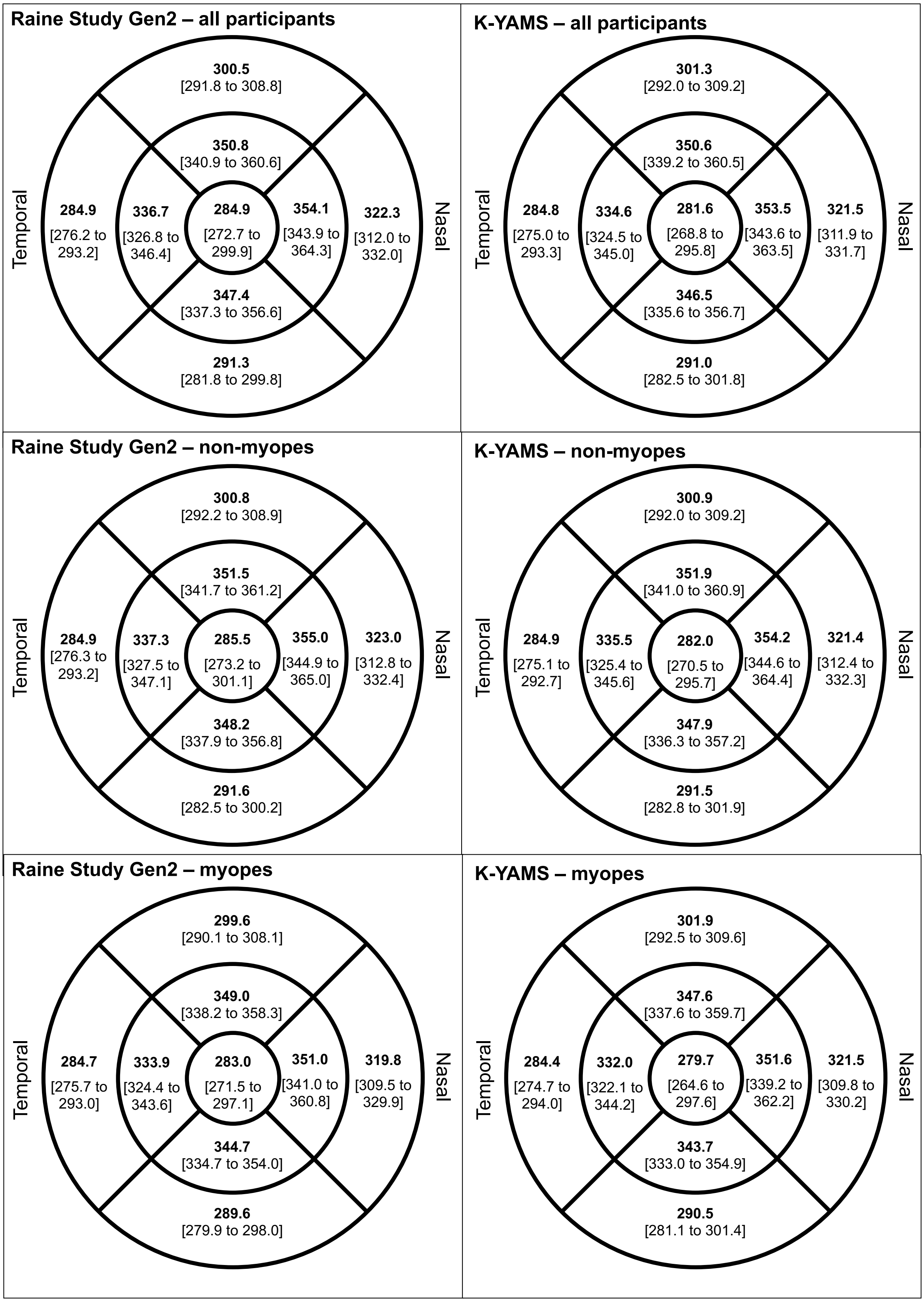

Supplement: S2 Fig — (PDF) [file pone.0266909.s002.pdf]
